# Proprietary Preparations

**Published:** 1908-01-25

**Authors:** 


### Editor's Letter-Box.

### PROPRIETARY PREPARATIONS.

Sir,—I read with much pleasure your temperate and well-reasoned leading article regarding such in The Hospital of January 18. There is one point which cannot be too strongly emphasised, and that is that too great care cannot be exercised by medical practitioners in prescribing proprietary preparations. I venture to say that there are few who would be so bigoted as to take up the attitude that proprietary preparations should never be prescribed. I, for my part, should not hesitate to prescribe any drug, be it old or new, if I thought it would benefit my patients; but I maintain that it should only be prescribed under its technical name. I frequently employ many of the newer remedies, but I use only those the compositions of which are known. Let a drug or a combination of drugs be known by any convenient name in private intercourse between medical men and pharmacists or among the medical profession itself, but in the prescription that is to be handed to the patient let the full technical designation or the full formula be written. If this were more generally carried out, then the use by unskilled persons of many remedies, which have become almost universally known, though not universally understood, would be to a certain extent checked. That some members of the public have become their own unqualified doctors is partly due to the medical profession itself, and it behoves the profession to counteract this dangerous tendency.

As you truly say in the leading article already referred to, "both medicine and pharmacy are progressive arts," and it would indeed be a pity if further developments were to be hindered by certain abuses which undoubtedly have arisen, but which might be checked. I am, etc.,

Alpha.

